# Fit Accuracy of Plate-Type Retainers Fabricated Using Dental CAD/CAM Systems: A Controlled In Vitro Comparison Using Typodont-Derived Models

**DOI:** 10.3390/dj13110487

**Published:** 2025-10-23

**Authors:** Kosuke Nomi, So Koizumi, Masatoshi Shimura, Kazuhide Seimiya, Osamu Nikaido, Heetae Park, Reina Hatanaka, Masahiro Takahashi, Shinya Fuchida, Tetsutaro Yamaguchi

**Affiliations:** 1 Department of Orthodontics, School of Dentistry, Kanagawa Dental University, Yokosuka, Kanagawa 238-8580, Japan; koizumi@kdu.ac.jp (S.K.); nikaido@kdu.ac.jp (O.N.); park@kdu.ac.jp (H.P.); hatanaka@kdu.ac.jp (R.H.); takahashi.masahiro@kdu.ac.jp (M.T.); t.yamaguchi@kdu.ac.jp (T.Y.); 2 Division of Dental Technology, Department of Dental Clinical Support, School of Dentistry, Kanagawa Dental University, Yokosuka, Kanagawa 238-8580, Japan; m.shimura@kdu.ac.jp (M.S.); seimiya@kdu.ac.jp (K.S.); 3 Department of Education Planning, School of Dentistry, Kanagawa Dental University, Yokosuka, Kanagawa 238-8580, Japan; fuchida@kdu.ac.jp

**Keywords:** conventional retainer, CAD/CAM retainer, impression replica technique, 3D triple-scan protocol, fit accuracy

## Abstract

**Objectives:** This study aimed to compare the fit accuracy between retainers fabricated using conventional cold-curing resin (hereinafter referred to as “conventional retainers”) and those fabricated using three-dimensional (3D) printing based on computer-aided design/computer-aided manufacturing (CAD/CAM) technology (hereinafter referred to as “CAD/CAM retainers”). Furthermore, the study aimed to compare two different methods to evaluate the fit accuracy: the impression replica technique and the 3D triple-scan protocol. **Methods:** For each of the 20 working models derived from a maxillary typodont, one conventional retainer and one CAD/CAM retainer were fabricated. The fit accuracy was evaluated using the impression replica technique and the 3D triple-scan protocol. Measurements were taken at 12 points on each model, and the differences in thickness (gap) were analyzed using Wilcoxon’s signed-rank test. Moreover, the correlation between thickness and measurement site was evaluated using Spearman’s rank correlation coefficient. **Results:** In both evaluation methods, the CAD/CAM retainers exhibited superior fit accuracy compared to the conventional retainers. Notably, the 3D triple-scan protocol clearly demonstrated that the fit accuracy differed depending on the measurement site. **Conclusions:** CAD/CAM retainers demonstrated superior fit accuracy compared to conventional retainers, possibly because digital design can account for polymerization shrinkage. In the impression replica technique, the median (interquartile range) thickness for the conventional retainers was 0.169 (0.120–0.260) mm, whereas that for the CAD/CAM retainers was 0.136 (0.096–0.198) mm. The CAD/CAM retainers showed significantly smaller gap values (*p* < 0.001). Within the limitations of this in vitro study, CAD/CAM retainers showed significantly smaller gap values than conventional retainers, indicating improved fit accuracy. In particular, the 3D triple-scan protocol accurately captured site-specific variations in fit accuracy among the anterior, canine, and molar regions.

## 1. Introduction

In recent years, the use of computer-aided design/computer-aided manufacturing (CAD/CAM) technologies in the field of orthodontics has expanded rapidly [1,2,3].

Traditionally, dental models have been fabricated using dental plaster, regarded as the “gold standard” due to its affordability, ease of use, and ability to accurately reproduce fine anatomical details of hard and soft tissues, such as occlusal morphology and the gingival margin [4].

With the advancement of intraoral scanners and digital modeling, CAD/CAM technology has become more prevalent in orthodontics. The development of cost-effective three-dimensional (3D) printers and biocompatible resins has made 3D printing feasible and increasingly popular in clinical orthodontics [5]. Compared to subtractive methods, additive manufacturing (e.g., 3D printing) offers higher precision in reproducing undercuts and complex geometries with reduced material waste and the ability to rapidly produce large, customized products [6].

In orthodontics, plaster models have been used not only to acquire intraoral data but also to fabricate functional appliances, retention devices, and expanders (including tooth-borne, bone-borne, and hybrid types). The use of 3D printing to fabricate such appliances is anticipated to become more common in the future.

After the active phase of orthodontic treatment, conventional plate-type retainers, such as those of the Hawley type, are used to maintain tooth alignment and promote tissue remodeling. These retainers are also fabricated on plaster models. The base of a conventional retainer is typically formed by layering monomer liquid and polymer powder directly onto the working model using the cold-curing (layering) method. Plate-type retainers possess unique clinical characteristics that distinguish them from clear thermoplastic retainers. The cold-cured acrylic resin used for these retainers exhibits relatively small polymerization shrinkage and low residual monomer content, resulting in superior long-term dimensional stability. Because of these material-specific factors, plate-type retainers require an independent evaluation of fit accuracy distinct from that of clear retainers.

Resin 3D printers based on stereolithography offer benefits such as reduced residual monomer content and improved dimensional stability due to their manufacturing process. Several studies have confirmed that dental models fabricated with resin 3D printers are clinically suitable [7,8,9].

As retainers are in prolonged contact with tooth and mucosal surfaces, similarly to dentures and crowns, high fit accuracy is essential. If CAD/CAM retainers fabricated using resin 3D printers can achieve high fit accuracy, this may shorten technician labor time and enhance patient satisfaction. Moreover, in the event of loss or damage, identical retainers can be easily reproduced from the CAD data, and adjustments can be made digitally. Given these advantages, retainers fabricated using CAD/CAM are expected to become more prevalent. However, to replace conventional retainers, CAD/CAM retainers must demonstrate equivalent or superior fit accuracy.

To date, studies on the fit accuracy of retainers in orthodontics have primarily focused on clear retainers fabricated using 3D printing. Various methods include pressure-molded clear retainers on printed models and retainers fabricated directly from intraoral scan data without models. For instance, Cole et al. measured the distance between the model and the internal surface of clear retainers via digital scanning. They reported that 3D-printed retainers showed inferior fit accuracy compared to conventional ones, but the discrepancies remained within a clinically acceptable range (≤0.5 mm) [10]. ElShebiny et al. superimposed the digital data of 3D-printed retainers onto master files and analyzed deviations using 3D imaging software: they concluded that both SLA and DLP technologies could produce retainers with clinically acceptable accuracy [11].

In this study, we aimed to compare the fit accuracy between conventional retainers fabricated using the layering method and CAD/CAM retainers fabricated using resin 3D printing technology. Furthermore, the study aimed to compare two different methods to evaluate the fit accuracy. Recent bibliometric research has indicated that 3D printing is the second most frequently studied digital technology in orthodontics among impactful publications. This finding underscores the importance of 3D printing in orthodontic research and further emphasizes the significance of evaluating its clinical applicability in retainer fabrication [12].

## 2. Materials and Methods

### 2.1. Fabrication of Working Models

A maxillary typodont model was used as the master model to fabricate both conventional and CAD/CAM retainers. Impressions were taken using silicone impression material (Examixfine, GC Corporation, Tokyo, Japan), and 20 working models were duplicated using extra-hard dental stone (New Fujirock, GC Corporation, Tokyo, Japan). One conventional and one CAD/CAM retainer were fabricated for each working model. After reviewing previous similar studies, the effect size was set to 0.8, the significance level to 0.05, and the statistical power to 0.8, resulting in a sample size of 20 pairs [13]. The assumed effect size (d = 0.8) was determined as a pragmatic estimate by referencing previous studies on the fit accuracy of removable dental appliances, including prosthodontic and orthodontic retainers, which generally reported large practical differences between fabrication methods. Although the study by Emera et al. (2022) focused on complete dentures, it employed a comparable statistical framework—a two-group comparison under nonparametric conditions with an assumed large effect size (d = 0.8)—which supports the rationale for the present sample size estimation. To minimize procedural errors, all procedures were performed by a single dentist throughout the study [13].

### 2.2. Fabrication of Conventional Retainers

Conventional retainers were fabricated by applying orthodontic resin material (Orthopalate, Shofu Inc., Kyoto, Japan) to the working model via the layering technique. This technique involves sprinkling polymer powder onto the model and then repeatedly applying a monomer liquid to increase the resin area and achieve the desired thickness. After layering, polymerization was performed using a pressure polymerizer (water temperature: 44–46 °C; pre-polymerization time: 5 min; main polymerization time: 30 min). Before layering, a denture base resin separator (AcrSep, GC Corporation, Tokyo, Japan) was applied to the working model.

### 2.3. Fabrication of CAD/CAM Retainers

CAD/CAM retainers were designed by scanning the working model using a model scanner (UP360+, Up3D) to obtain STL data. The resin base was digitally designed using CAD software (Dental Wings Open System, Dental Wings Inc., Montreal, QC, Canada; available at: https://www.dentalwings.com/, accessed on 8 September 2025). The design was then converted into printable data using CAM software (ChituBox, Shenzhen CBD Technology Co., Ltd., Shenzhen, China; available at: https://www.chitubox.com/, accessed on 8 September 2025). During the digital design process, a manufacturer-recommended pre-compensation ratio of 100.60% was applied to account for expected polymerization shrinkage. The retainers were fabricated via stereolithography using a 3D printer (DH Sonic Mighty 4K, Denken High Dental Co., Ltd., Kyoto, Japan) with a 3D printer resin (DH Print Sprint & Guide, Denken High Dental Co., Ltd., Kyoto, Japan). Printing was performed at room temperature and with a layer thickness of 0.05 mm. All fabricated CAD/CAM retainers were thoroughly washed with isopropyl alcohol (IPA) to remove any nonpolymerized resin, followed by air-drying. After fabrication, post-curing was performed in a light-curing unit for 30 min under a wavelength range of 375–495 nm.

### 2.4. Evaluation Using the Impression Replica Technique

The fit accuracy was evaluated using two methods: the impression replica technique with silicone-based fit-checking material [14,15,16,17] and the 3D triple-scan protocol using a model scanner [18,19,20]. In the impression replica technique, silicone-based fit-checking material (Fit Checker II, GC Corporation, Tokyo, Japan) was applied to the tissue surface of the retainer and pressed onto the working model (pressure: approximately 10 kg; time: 3 min 30 s). Subsequently, impressions—including the retainer and silicone material—were taken using alginate impression material (Aroma Fine Plus, GC Corporation, Tokyo, Japan). The inner surface of the retrieved impression was filled with hydrophilic vinyl silicone impression material (Examixfine Injection Type, GC Corporation, Tokyo, Japan) and then covered with putty-type vinyl silicone impression material (Exafine Putty Type, GC Corporation, Tokyo, Japan) to produce a silicone replica. The applied pressure (~10 kg) was standardized by placing the working model on a weighing scale and pressing the retainer from above until the scale indicated approximately 10 kg, which was then maintained for 3 min 30 s. The applied pressure (~10 kg) was determined on the basis of the first author’s clinical experience, aiming to ensure complete seating of the retainer and achieve uniform thickness of the silicone fit-checking material. As the literature lacks established guidelines defining the optimal load, this empirically determined value was used to maintain consistency throughout the experiments. To ensure stability, the pressure was applied mainly around the canine region; therefore, uniform pressure across the entire plate could not be achieved.

**Figure 1:**
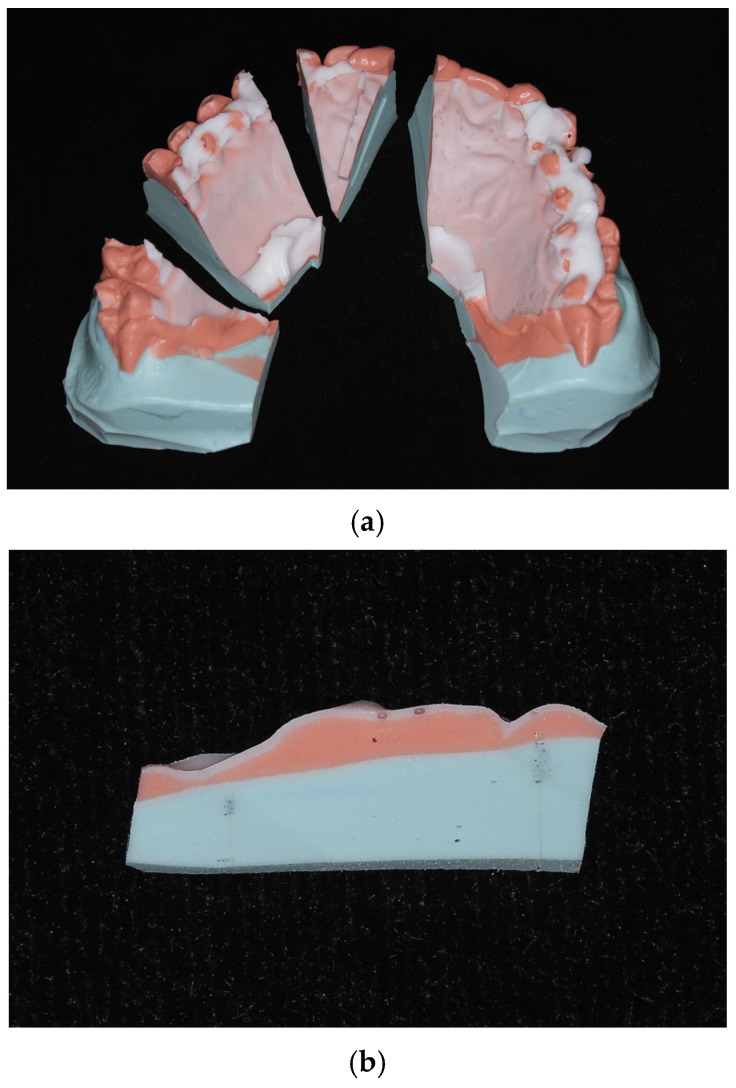
(**a**) Impression replica. (**b**) Sectioned impression replica.

Measurement points included the tooth surfaces (1.0 mm above the cervical margin) and tissue surfaces (10.0 mm below the cervical margin for central incisors and canines, 7.0 mm below for first molars) of the bilateral maxillary central incisors, canines, and first molars—a total of 12 locations. The replicas were sectioned at each measurement site. The thickness of the silicone fit-checking material was measured using a desktop scanning electron microscope (JCM-6000Plus, JEOL Ltd., Tokyo, Japan). Each site was measured three times, and the average was recorded (Figure 1a,b).

### 2.5. Evaluation Using a 3D Triple-Scan Protocol

**Figure 2:**
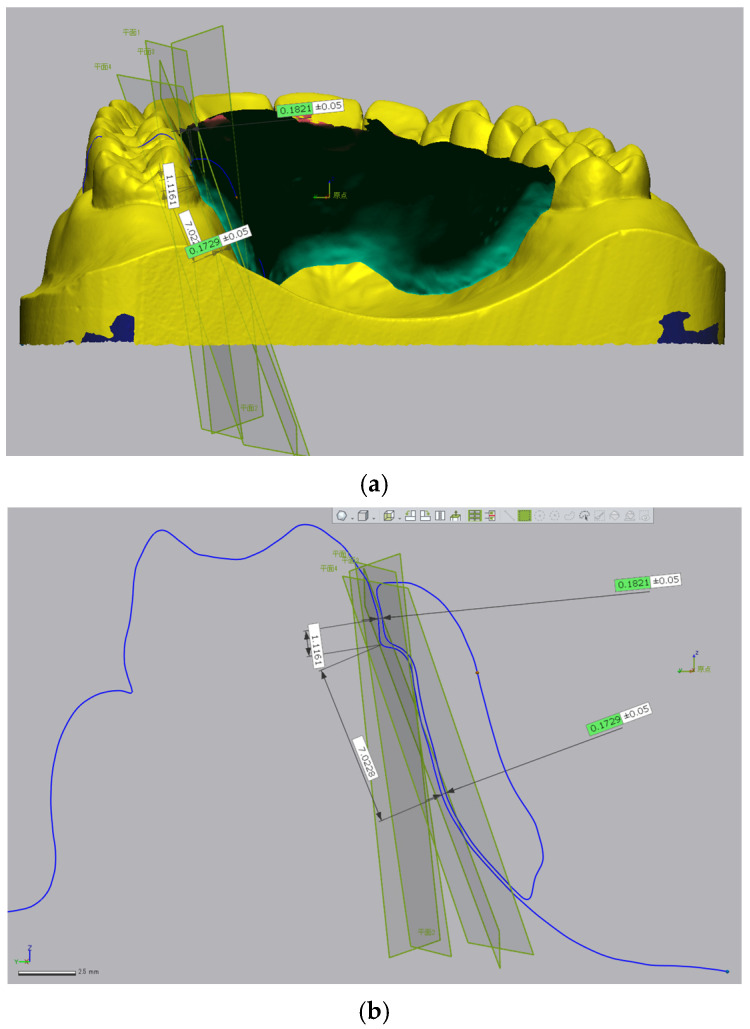
(**a**) Alignment of the model and retainer in Geomagic Control X. (**b**) Gap measurement in Geomagic Control X. The Japanese terms “平面” (plane) and “原点” (origin) indicate the reference surface and the coordinate origin, respectively.

In the 3D triple-scan protocol, the working model and the retainer were each scanned individually using a model scanner (UP360+, Up3D Technology Co., Ltd., Shenzhen, China). Subsequently, the retainer was seated on the working model and rescanned. Prior to scanning, the polished surface of the retainer was sandblasted, and scanning powder was sprayed on the tissue surface to reduce light reflection. The model and retainer surfaces were lightly sandblasted and coated with scanning powder to reduce reflectivity. The scanning powder used had a particle size of approximately 0.003 mm, which is negligible relative to the overall measurement scale. These scan data were superimposed using 3D image analysis software (Geomagic Control X, v2018.1.2; 3D Systems, Inc., Rock Hill, SC, USA), and the gap between the model surface and the tissue surface of the retainer was measured (Figure 2a,b). The measurement points were the same as those used in the impression replica method. In the triple-scan workflow, we did not propagate landmarks via a shared coordinate system. Instead, the same 12 sites were re-identified on each superimposed mesh using a rule-based landmarking strategy in Geomagic Control X. Tooth-surface points were placed 1.0 mm coronal to the cervical margin (mid-buccal) of the bilateral first molars, canines, and central incisors, and tissue-surface points were placed 10.0 mm apical for incisors/canines and 7.0 mm for first molars relative to the cervical margin, following the same definitions used for the replica sections.

All landmarks were placed by a single operator using the software’s distance/offset and point tools to harmonize site definitions between the two methods.

The scanned datasets were aligned using the best-fit alignment function implemented in Geomagic Control X (3D Systems). The alignment tolerance and scanner calibration accuracy were not quantitatively verified in this study; however, visual inspection confirmed correct registration without noticeable deviation. General specifications for laboratory optical scanners indicate that measurement precision is typically within several tens of micrometers. In addition, because no direct agreement or calibration analysis was performed between the two evaluation methods, the differences observed between the impression replica and triple-scan results should be interpreted as methodological rather than as true precision discrepancies. Nevertheless, the repeatability of each measurement method was preliminarily verified through intra-operator reliability assessment using intraclass correlation coefficients (ICCs) and Dahlberg’s formula, confirming acceptable reproducibility [21]. However, the detailed numerical results are not shown because this study’s focus was on comparative tendencies rather than absolute precision metrics. The present comparison was intended to descriptively highlight systematic tendencies between the two techniques rather than to establish an absolute measurement standard.

### 2.6. Classification and Integration of Measurement Sites

To evaluate the relationship between gap thickness and anatomical region, the measurement sites were classified into three areas: “incisor region,” “canine region,” and “molar region.” Data from the right and left sides were integrated accordingly. For example, the values for 16A, 16B, 26A, and 26B were combined to represent the “molar region”.

### 2.7. Statistical Analysis

The normality of the data was assessed using the Shapiro–Wilk test. Since normality was not confirmed, nonparametric tests were used. Wilcoxon’s signed-rank test was employed to compare gap thicknesses between the two fabrication methods (conventional vs. CAD/CAM retainers) and between the two evaluation methods (impression replica vs. 3D triple-scan protocol).

In addition, Spearman’s rank correlation coefficient was used to assess the relationship between gap thickness and anatomical regions (incisor, canine, and molar). The variable “region” (incisor, canine, and molar) was treated as a pseudo-ordinal factor to explore whether gap thickness exhibited a monotonic trend from anterior to posterior regions. This approach was exploratory and aimed to identify general tendencies rather than to imply strict linearity or geometric continuity. All statistical analyses were performed using statistical software (IBM SPSS Statistics v28.0.1.0; IBM Corp., Armonk, NY, USA), and a significance level of 0.05 was adopted.

## 3. Results

### 3.1. Results of the Impression Replica Technique

To compare the fit accuracy between the 20 conventional and 20 CAD/CAM retainers, the differences in the thickness of the fit-checking material were evaluated using the impression replica technique. The median (interquartile range) thickness for the conventional retainers was 0.169 (0.120–0.260) mm, whereas that for the CAD/CAM retainers was 0.136 (0.096–0.198) mm (Table 1). The CAD/CAM retainers showed significantly lower thickness values (*p* < 0.001).

**Figure 3:**
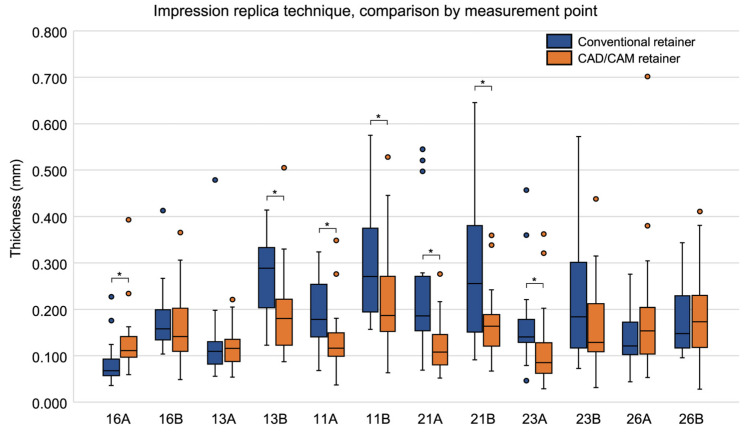
Box plots comparing the thickness of silicone fit-checking material at each measurement site in conventional and CAD/CAM retainers, measured using the impression replica technique. Medians and interquartile ranges are shown. Asterisks (*) indicate measurement sites with significant differences between groups (Wilcoxon’s signed-rank test, *p* < 0.05).

When comparing thickness by measurement site, significant differences between the conventional and CAD/CAM retainers were observed at seven sites: 16A (*p* = 0.003), 13B (*p* = 0.008), 11A (*p* = 0.009), 11B (*p* = 0.030), 21A (*p* < 0.001), 21B (*p* = 0.009), and 23A (*p* = 0.007). At six of these sites—13B, 11A, 11B, 21A, 21B, and 23A—the CAD/CAM retainers had significantly smaller thickness values (Table 2, Figure 3). Table 2 summarizes the statistically significant sites, while the full dataset for all 12 measurement sites is presented in the Supplementary Material (Table S1).

### 3.2. Results of the 3D Triple-Scan Protocol

To compare the fit accuracy using the 3D triple-scan protocol, the gap between the model surface and the tissue surface of the retainer was measured in the 20 conventional and 20 CAD/CAM retainers. The median (interquartile range) gap for conventional retainers was 0.206 (0.131–0.315) mm, whereas that for CAD/CAM retainers was 0.182 (0.091–0.289) mm (Table 1). The CAD/CAM retainers exhibited significantly smaller gap values compared to the conventional retainers (*p* = 0.013).

**Figure 4:**
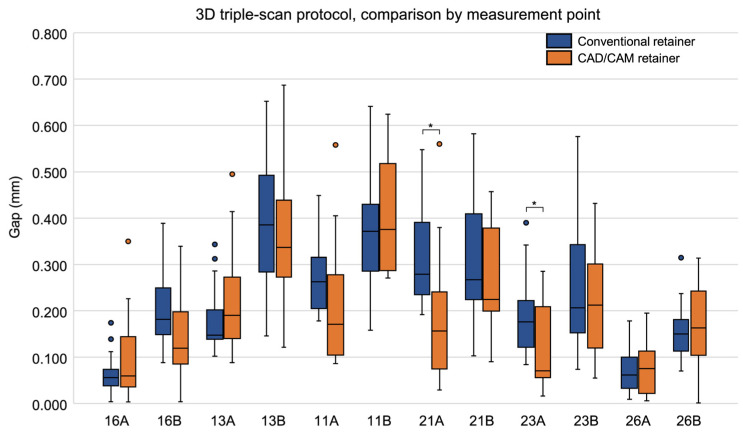
Box plots comparing the gap (distance) between the model surface and the tissue surface of the retainer at each measurement site in conventional and CAD/CAM retainers, measured using the 3D triple-scan protocol. Medians and interquartile ranges are shown. Asterisks (*) indicate measurement sites with significant differences between groups (Wilcoxon’s signed-rank test, *p* < 0.05). CAD/CAM, computer-aided design/computer-aided manufacturing.

When comparing gaps by measurement site, significant differences between the two groups were observed at two sites: 21A (*p* = 0.002) and 23A (*p* = 0.028), with CAD/CAM retainers showing significantly smaller gaps at both locations (Table 3, Figure 4). The complete dataset for all 12 measurement sites is available in the Supplementary Material (Table S2).

### 3.3. Comparison Between the Two Evaluation Methods

For the 20 conventional retainers, the impression replica technique showed a median (interquartile range) thickness of 0.169 (0.120–0.260) mm, whereas the 3D triple-scan protocol showed a median gap of 0.206 (0.131–0.315) mm (Table 4). The impression replica technique yielded significantly smaller values (*p* < 0.001). Similarly, for the 20 CAD/CAM retainers, the median thickness measured using the impression replica technique was 0.136 (0.096–0.198) mm, whereas the 3D triple-scan protocol showed 0.182 (0.091–0.289) mm (Table 4), again with significantly smaller values observed in the impression replica technique (*p* < 0.001).

**Figure 5:**
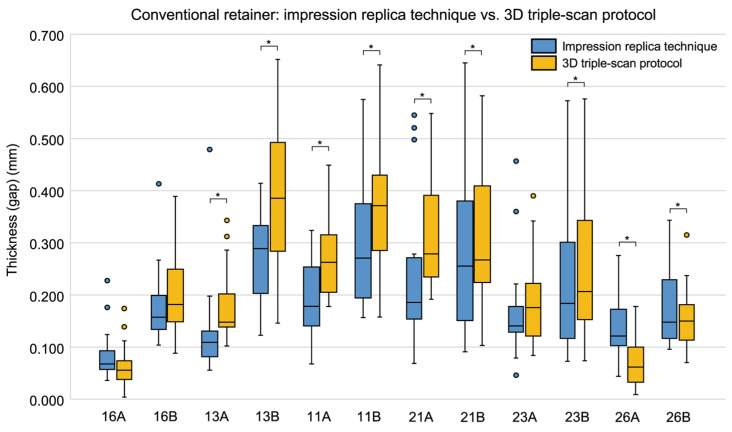
Box plots comparing the thickness of silicone fit-checking material in conventional retainers measured using the impression replica technique and the gap (distance) between the model surface and the tissue surface of the same retainers measured using the 3D triple-scan protocol. Medians and interquartile ranges are shown. Asterisks (*) indicate measurement sites with significant differences between methods (Wilcoxon’s signed-rank test, *p* < 0.05).

When comparing results at individual measurement sites for the conventional retainers, significant differences between the two methods were observed at nine sites: 13A (*p* = 0.002), 13B (*p* < 0.001), 11A (*p* < 0.001), 11B (*p* = 0.004), 21A (*p* < 0.001), 21B (*p* = 0.037), 23B (*p* = 0.005), 26A (*p* < 0.001), and 26B (*p* = 0.015; Table 5, Figure 5). Among these, eight sites—13A, 13B, 11A, 11B, 21A, 21B, 23B, and 26B—showed significantly smaller thickness values with the impression replica technique.

**Figure 6:**
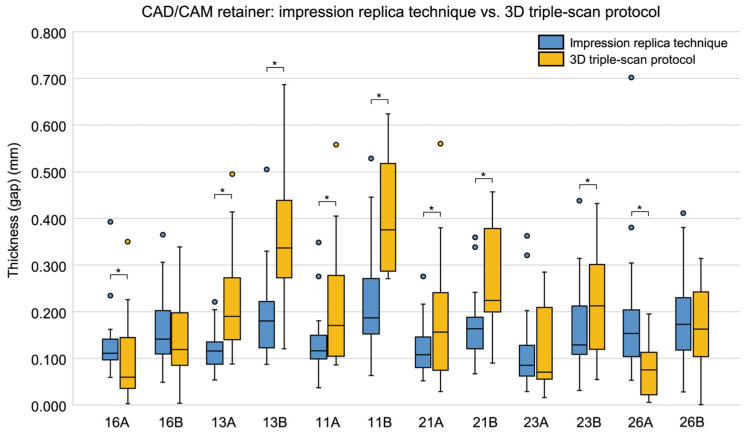
Box plots comparing the thickness of silicone fit-checking material in CAD/CAM retainers measured using the impression replica technique and the gap (distance) between the model surface and the tissue surface of the same retainers measured using the 3D triple-scan protocol. Medians and interquartile ranges are shown. Asterisks (*) indicate measurement sites with significant differences between methods (Wilcoxon’s signed-rank test, *p* < 0.05).

For CAD/CAM retainers, significant differences between the two methods were observed at nine sites: 16A (*p* = 0.002), 13A (*p* < 0.001), 13B (*p* < 0.001), 11A (*p* = 0.001), 11B (*p* < 0.001), 21A (*p* = 0.014), 21B (*p* < 0.001), 23B (*p* = 0.017), and 26A (*p* < 0.001; Table 6, Figure 6). Of these, seven sites—13A, 13B, 11A, 11B, 21A, 21B, and 23B—showed significantly smaller values in the impression replica technique.

### 3.4. Correlation Between Thickness (Gap) and Measurement Region

**Figure 7:**
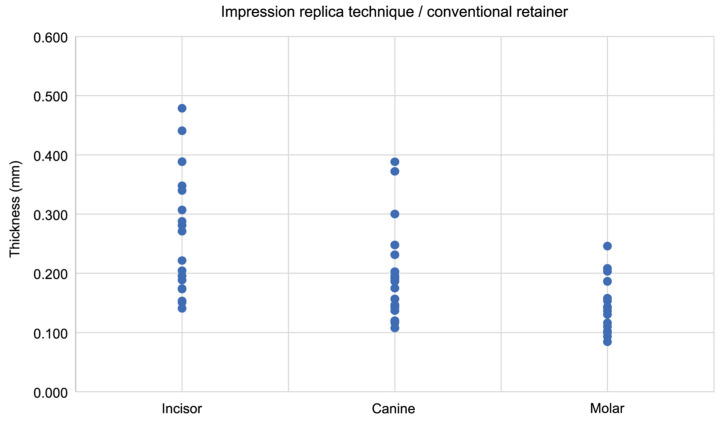
The scatter plot shows the correlation between the thickness of silicone fit-checking material and the measurement site in conventional retainers, as measured using the impression replica technique. Analysis was performed using Spearman’s rank correlation coefficient.

**Figure 8:**
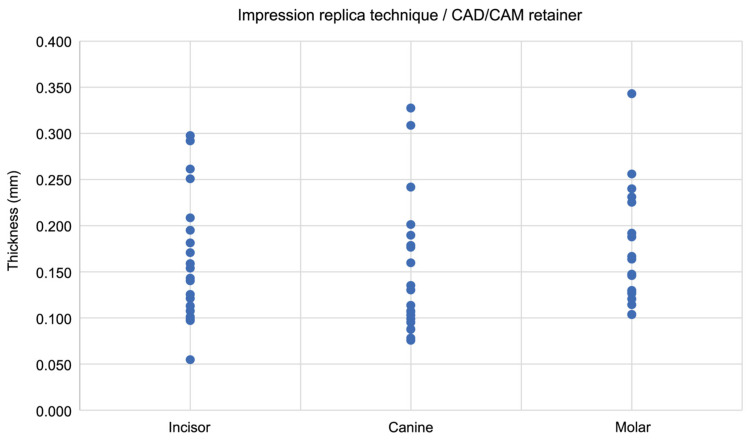
The scatter plot shows the correlation between the thickness of silicone fit-checking material and the measurement site in CAD/CAM retainers, as measured using the impression replica technique. Analysis was performed using Spearman’s rank correlation coefficient.

The correlation between gap thickness and measurement region was evaluated in both the impression replica technique and the 3D triple-scan protocol. For the conventional retainers evaluated using the impression replica technique, a significant moderate negative correlation was found, indicating that the thickness decreased from incisor to molar regions (Spearman’s ρ = −0.513, *p* < 0.001; Figure 7). In contrast, no significant correlation was observed for the CAD/CAM retainers (Spearman’s ρ = 0.062, *p* = 0.635; Figure 8).

**Figure 9:**
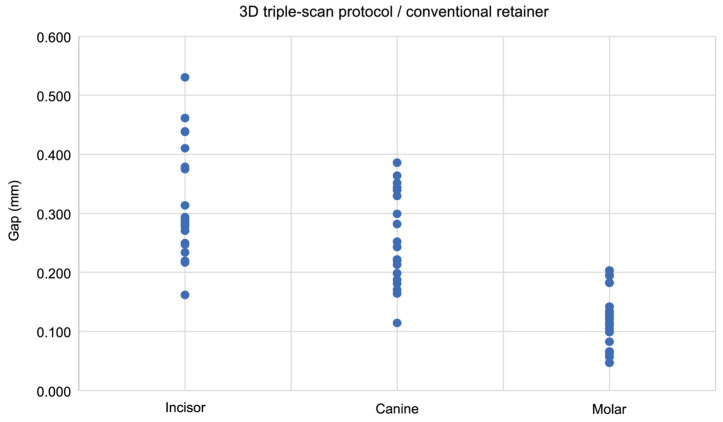
The scatter plot shows the correlation between the gap (distance) between the model surface and the tissue surface and the measurement site in conventional retainers, as measured using the 3D triple-scan protocol. Analysis was performed using Spearman’s rank correlation coefficient.

**Figure 10:**
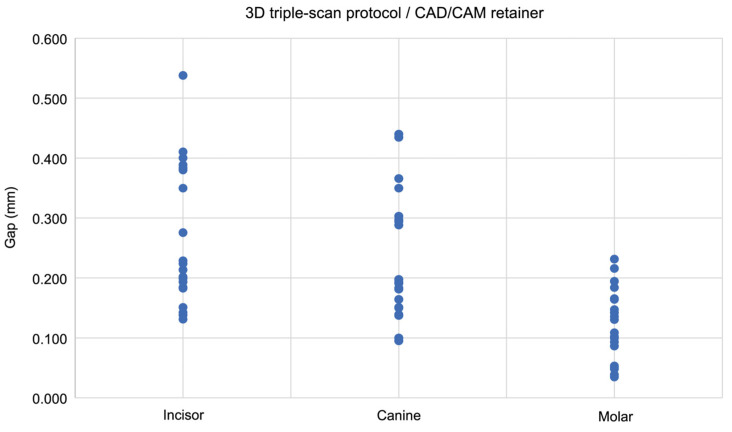
The scatter plot shows the correlation between the gap (distance) between the model surface and the tissue surface and the measurement site in CAD/CAM retainers, as measured using the 3D triple-scan protocol. Analysis was performed using Spearman’s rank correlation coefficient.

The 3D triple-scan protocol indicated a significant strong negative correlation in the conventional retainers, showing decreasing thickness values from incisor to molar regions (Spearman’s ρ = −0.756, *p* < 0.001; Figure 9). A significant moderate negative correlation was found in CAD/CAM retainers (Spearman’s ρ = −0.555, *p* < 0.001; Figure 10).

## 4. Discussion

In dental clinical practice, evaluating the fit accuracy of intraoral devices—such as dentures, prostheses, and removable orthodontic appliances—is critically important to ensure the highest possible precision in their design and fabrication. Among the various methods, using calipers to measure the thickness of silicone fit-checking materials is one of the most widely adopted techniques for assessing the accuracy of denture bases [22]. However, because of the elasticity of silicone-based materials, accurate thickness measurements can be challenging. Therefore, in this study, a non-contact desktop scanning electron microscope was used to determine the thickness. Additionally, a second non-contact method—the 3D triple-scan protocol—was employed. This method evaluates the fit accuracy by measuring the gap between the model surface and the retainer’s tissue surface, rather than relying on the thickness of silicone materials. To the best of our knowledge, no previous studies have investigated the fit accuracy of retainers using both the impression replica technique and the 3D triple-scan protocol.

One of the greatest challenges in orthodontics is achieving long-term stability of treatment outcomes [23]. Past studies have identified factors such as proper cusp interdigitation and maintenance of intercanine width as contributing to stability. However, more recent research indicates that the most critical factor in ensuring long-term post-treatment stability is the use of retainers [24,25,26]. Hawley and vacuum-formed retainers are the most commonly used retention devices [27,28].

In this study, we compared the fit accuracy of conventional retainers fabricated using the layering method and CAD/CAM retainers fabricated using resin 3D printers. The results demonstrated that the CAD/CAM retainers exhibited significantly smaller thickness (gap) values in both evaluation methods, indicating their superior fit accuracy. In the 3D triple-scan protocol, the CAD/CAM retainers showed significantly smaller values at 21A and 23A, which correspond to the anterior and canine regions that are esthetically and functionally important. The smaller values observed in these regions suggest superior morphological adaptability achieved through CAD/CAM fabrication, which may contribute to reduced gingival irritation and improved long-term fit stability. However, at certain measurement sites, such as 16A in the impression replica results, conventional retainers exhibited smaller median gap values than CAD/CAM retainers.

Although CAD/CAM retainers exhibited significantly smaller median gap values than conventional retainers, the absolute differences were small (approximately 0.03 mm) and well within the clinically acceptable range of 0.25–0.50 mm. Therefore, while the CAD/CAM method demonstrated higher reproducibility and uniformity, both fabrication methods can be regarded as clinically acceptable in terms of fit accuracy. Although the clinical thresholds (0.25 mm and 0.5 mm) were not visually indicated in the box plots due to layout constraints, the majority of the measured values were below these limits, supporting the interpretation that the fit of both retainer types was clinically acceptable.

These localized reversals are considered to reflect site-specific morphological or methodological variability rather than a systematic deviation from the overall trend, as the pooled data consistently favored the CAD/CAM retainers. These findings suggest that CAD/CAM retainers may serve as viable alternatives to conventional retainers.

Our findings are consistent with those of Cole et al., who compared the accuracy of clear retainers fabricated using three methods: traditional vacuum forming, commercial vacuum forming, and 3D printing. Although the retainers made using traditional vacuum forming showed the highest fit accuracy with the model, 3D-printed clear retainers also had clinically acceptable accuracy [10]. Although a standardized reference for the maximum acceptable gap between a retainer and the model surface currently does not exist, some studies suggest that discrepancies within 0.5 mm are clinically acceptable. For instance, Camardella et al. and Sherman et al. reported that deviations within ±0.5 mm in 3D-printed models are tolerable in clinical use [29,30]. On the other hand, Boo et al. suggested that a clinical threshold of 0.25 mm is more appropriate for clear retainers, as gaps exceeding this value may increase the risk of relapse due to insufficient contact between the retainer and teeth [31,32]. Therefore, a maximum gap of 0.25 mm is often considered the standard in the fabrication of 3D-printed clear retainers. Based on the literature, we concluded that a gap within the range of 0.25 to 0.50 mm can be considered clinically acceptable. However, these thresholds were mainly derived from studies on clear retainers and 3D-printed models. To date, no established clinical acceptability threshold has been defined specifically for plate-type acrylic retainers; therefore, these values were used in this study as reference indicators for comparative discussion.

A survey investigating the use of 3D printing technology in orthodontic practices in North America—where orthodontics is highly advanced—reported that approximately 75% of orthodontists use 3D printing in some form, suggesting that its application will continue to grow [33].

From the perspective of polymerization shrinkage, the layering method offers several advantages: ease of manipulation, ability to adjust the base shape and thickness, and esthetic customization by layering resins of different colors or translucencies. The layering method also has a lower shrinkage rate (0.2%) than the heat-curing method (0.3–0.5%), suggesting better dimensional stability. However, compared to heat-cured resins, layered resin contains about ten times more residual monomers (3–5%), resulting in inferior mechanical properties and greater water absorption and discoloration [34]. Although the shrinkage rate during curing is reported to be 0.2%, it remains unclear whether resin is consistently mixed in precise ratios during clinical fabrication. In addition, the conventional layering technique inherently involves the manual application of resin, which may introduce operator-dependent variability in base thickness and uniformity. This potential variability should be recognized as a limitation of the conventional fabrication method.

In contrast, 3D printers allow for pre-compensation—that is, dimensional adjustments made during the digital design phase to account for expected polymerization shrinkage during curing. This feature, not available in the conventional method, enables highly accurate production [35]. In the present study, pre-compensation was performed using a manufacturer-recommended compensation ratio (100.60%), and the retainer was fabricated using stereolithography. This pre-compensation likely contributed to the high fit accuracy of the CAD/CAM retainers, although this effect was not experimentally verified in this study. This pre-compensation setting was applied during the digital design stage, as described in the Materials and Methods section.

Interestingly, the impression replica technique yielded smaller differences between corresponding measurement sites compared to the 3D triple-scan protocol. This discrepancy may stem from the difference between contact methods (with pressure) and non-contact methods (without pressure). In the impression replica technique, the retainer is pressed against the incisor portion of the plate (around the canine region), possibly resulting in less variation in gap thickness across the plate. Svanborg et al. noted that the pressure applied to the restoration during the impression replica method could result in tighter fits compared to methods such as the 3D triple-scan protocol [19]. Hasanzade et al. also pointed out issues such as dimensional changes or tearing in silicone materials used in the impression replica method [20]. In the impression replica technique, pressure was manually applied using a weighing scale to approximate 10 kg for 3 min 30 s, mainly in the canine region. As a result, a completely uniform pressure distribution could not be ensured, which should be considered a limitation of this study. Furthermore, the load of approximately 10 kg applied during the impression replica procedure was empirically determined rather than standardized and was aimed at ensuring complete seating of the retainer. Future studies should explore a reproducible and clinically validated loading condition.

Furthermore, the 3D triple-scan protocol indicated a stronger negative correlation between region and thickness, indicating that the fit accuracy declines toward the incisor region. This suggests that the 3D triple-scan protocol may more accurately capture regional differences in fit. These results imply that non-contact methods such as the 3D triple-scan protocol are superior for an accurate fit evaluation, especially when uniform pressure across the entire plate is difficult to achieve in contact methods.

Conversely, despite the pressure involved in the impression replica technique, no significant correlation was observed between measurement site and thickness in CAD/CAM retainers. This suggests that CAD/CAM retainers may have a superior and more uniform fit than conventional retainers.

This study was conducted in vitro using a typodont, which cannot fully replicate actual intraoral conditions, such as saliva, temperature fluctuations, soft-tissue compliance, and insertion/removal forces. Therefore, future research involving real patients is necessary to comprehensively evaluate the fit accuracy, long-term clinical effectiveness, and patient comfort. In the 3D triple-scan protocol, a global best-fit registration algorithm implemented in Geomagic Control X (3D Systems) was used to align the scan data. However, the manufacturer’s detailed accuracy specification of the model scanner (UP360+, Up3D) could not be verified, and quantitative validation of scanner calibration and registration error was not performed.

Therefore, minor measurement uncertainty may have been introduced in the dimensional analysis. Furthermore, we acknowledge that no coordinate-based point transfer was used in the triple-scan workflow, which may leave a small degree of operator-dependent variability in landmark identification. Additionally, multiple site-level Wilcoxon tests were performed in an exploratory manner without adjustment for multiplicity, which may increase the risk of type I error, and results should be interpreted accordingly. Furthermore, as this study focused on relative rather than absolute differences, the statistical interpretation was primarily based on *p*-values. The lack of effect size and confidence interval reporting should be recognized as a limitation, and the observed differences should be interpreted in relative terms. In addition, the Spearman correlation analysis treated the categorical variable “region” (incisor/canine/molar) as pseudo-ordinal for exploratory purposes, which oversimplifies the complex three-dimensional geometry of the retainer base. Therefore, these correlations should be interpreted with caution. Furthermore, the print orientation and support structure during the 3D printing process were not strictly standardized across all specimens. This lack of uniformity may have introduced minor variability in dimensional accuracy among the fabricated retainers. Future studies should aim to standardize these parameters to further enhance reproducibility and precision; however, the rapid advancement of 3D printing technology is expected to further accelerate the development of digital orthodontics.

The evolution of 3D printing technology will likely lead to further reductions in manual fabrication steps, improved patient comfort through the production of more precise appliances, and shorter chairside time.

## 5. Conclusions

Within the limitations of this in vitro study, CAD/CAM retainers showed significantly smaller gap values than conventional retainers; these results suggest that CAD/CAM retainers have improved fit accuracy under these experimental conditions.

Although the silicone-based fit-checking materials used in the impression replica technique have been the standard method for evaluating the fit accuracy, this study indicates that non-contact methods—such as the 3D triple-scan protocol—may allow for more detailed and reproducible fit assessments, although their superiority in precision was not quantitatively verified.

## Figures and Tables

**Table 1:** Comparison of the median thickness (gap) values for 20 conventional retainers and 20 CAD/CAM retainers using the impression replica technique and the 3D triple-scan protocol.

Evaluation Method	Group	Median (IQR), mm	*p*-Value
Impression replica technique	Conventional retainers	0.169 (0.120–0.260)	―
	CAD/CAM retainers	0.136 (0.096–0.198)	<0.001 *
3D triple-scan protocol	Conventional retainers	0.206 (0.131–0.315)	―
	CAD/CAM retainers	0.182 (0.091–0.289)	0.013 *

* Significant difference (*p* < 0.05, Wilcoxon’s signed-rank test); 3D, three-dimensional; CAD/CAM, computer-aided design/computer-aided manufacturing; IQR, interquartile range.

**Table 2:** Comparison of the median thickness values at each measurement site between conventional and CAD/CAM retainers using the impression replica technique. Only statistically significant sites are shown. Detailed results for all 12 measurement sites are provided in the Supplementary Material (Table S1).

Measurement Region	Conventional Retainers Median (IQR), mm	CAD/CAM Retainers Median (IQR), mm	*p*-Value
16A	0.068 (0.057–0.107)	0.111 (0.095–0.144)	0.003 *
13B	0.289 (0.201–0.337)	0.180 (0.119–0.225)	0.008 *
11A	0.178 (0.138–0.261)	0.116 (0.094–0.150)	0.009 *
11B	0.271 (0.189–0.399)	0.187 (0.151–0.296)	0.030 *
21A	0.186 (0.145–0.273)	0.108 (0.076–0.146)	<0.001 *
21B	0.255 (0.151–0.384)	0.164 (0.121–0.191)	0.009 *
23A	0.141 (0.126–0.198)	0.085 (0.057–0.135)	0.007 *

* Significant difference (*p* < 0.05, Wilcoxon’s signed-rank test).

**Table 3:** Comparison of the median thickness (gap) values at each measurement site between conventional and CAD/CAM retainers using the 3D triple-scan protocol. Only statistically significant sites are shown. Detailed results for all 12 measurement sites are provided in the Supplementary Material (Table S2).

Measurement Region	Conventional Retainers Median (IQR), mm	CAD/CAM Retainers Median (IQR), mm	*p*-Value
21A	0.279 (0.232–0.401)	0.157 (0.066–0.273)	0.002 *
23A	0.176 (0.120–0.241)	0.071 (0.051–0.215)	0.028 *

* Significant difference (*p* < 0.05, Wilcoxon’s signed-rank test).

**Table 4:** Comparison of the median thickness (gap) values obtained using the impression replica technique and the 3D triple-scan protocol for 20 conventional and 20 CAD/CAM retainers.

Evaluation Method		Median (IQR), mm	*p*-Value
Conventional retainers	Impression replica technique	0.169 (0.120–0.260)	―
	3D triple-scan protocol	0.206 (0.131–0.315)	<0.001 *
CAD/CAM retainers	Impression replica technique	0.136 (0.096–0.198)	―
	3D triple-scan protocol	0.182 (0.091–0.289)	<0.001 *

* Significant difference (*p* < 0.05, Wilcoxon’s signed-rank test).

**Table 5:** Comparison of the median thickness (gap) values at each measurement site obtained using the impression replica technique and the 3D triple-scan protocol for conventional retainers.

Measurement Region	Impression Replica Technique Median (IQR), mm	3D Triple-Scan Protocol Median (IQR), mm	*p*-Value
16A	0.068 (0.057–0.107)	0.056 (0.035–0.075)	0.079
16B	0.158 (0.131–0.205)	0.182 (0.148–0.251)	0.167
13A	0.109 (0.080–0.136)	0.148 (0.138–0.222)	0.002 *
13B	0.289 (0.201–0.337)	0.386 (0.283–0.508)	<0.001 *
11A	0.178 (0.138–0.261)	0.263 (0.205–0.316)	<0.001 *
11B	0.271 (0.189–0.399)	0.372 (0.283–0.436)	0.004 *
21A	0.186 (0.145–0.273)	0.279 (0.232–0.401)	<0.001 *
21B	0.255 (0.151–0.384)	0.267 (0.216–0.424)	0.037 *
23A	0.141 (0.126–0.198)	0.176 (0.120–0.241)	0.455
23B	0.184 (0.105–0.309)	0.207 (0.150–0.344)	0.005 *
26A	0.121 (0.101–0.179)	0.062 (0.026–0.104)	<0.001 *
26B	0.148 (0.110–0.240)	0.150 (0.102–0.182)	0.015 *

* Significant difference (*p* < 0.05, Wilcoxon’s signed-rank test).

**Table 6:** Comparison of the median thickness (gap) values at each measurement site obtained using the impression replica technique and the 3D triple-scan protocol for CAD/CAM retainers.

Measurement Region	Impression Replica Technique Median (IQR), mm	3D Triple-Scan Protocol Median (IQR), mm	*p*-Value
16A	0.111 (0.095–0.144)	0.060 (0.027–0.158)	0.002 *
16B	0.142 (0.104–0.210)	0.119 (0.080–0.247)	0.145
13A	0.116 (0.087–0.140)	0.190 (0.138–0.281)	<0.001 *
13B	0.180 (0.119–0.225)	0.337 (0.268–0.455)	<0.001 *
11A	0.116 (0.094–0.150)	0.171 (0.104–0.303)	0.001 *
11B	0.187 (0.151–0.296)	0.376 (0.284–0.521)	<0.001 *
21A	0.108 (0.076–0.146)	0.157 (0.066–0.273)	0.014 *
21B	0.164 (0.121–0.191)	0.225 (0.197–0.386)	<0.001 *
23A	0.085 (0.057–0.135)	0.071 (0.051–0.215)	0.940
23B	0.129 (0.106–0.214)	0.213 (0.117–0.304)	0.017 *
26A	0.154 (0.099–0.232)	0.076 (0.019–0.114)	<0.001 *
26B	0.173 (0.116–0.236)	0.163 (0.098–0.248)	0.287

* Significant difference (*p* < 0.05, Wilcoxon’s signed-rank test).

## Data Availability

The data generated during this study are available from the corresponding author upon reasonable request.

## Supplementary Materials

The following supporting information can be downloaded at: https://www.mdpi.com/article/10.3390/dj13110487/s1, Table S1: Full dataset of median thickness (gap) values at all 12 measurement sites obtained using the impression replica technique; Table S2: Full dataset of median thickness (gap) values at all 12 measurement sites obtained using the 3D triple-scan protocol.

## Abbreviations

The following abbreviations are used in this manuscript:

3D	three-dimensional
CAD/CAM	computer-aided design/computer-aided manufacturing
