# Supplementary material for: Fit Accuracy of Plate-Type Retainers Fabricated Using Dental CAD/CAM Systems: A Controlled In Vitro Comparison Using Typodont-Derived Models
Source: Dent J (Basel). 2025 Oct 23;13(11):487. doi: 10.3390/dj13110487 (PMC12651359; doi:10.3390/dj13110487)
Supplement: Supplementary file 1 [file dentistry-13-00487-s001.zip › dentistry-3892804-supplementary.pdf]

Table S1. Comparison of the median thickness values at each measurement site between conventional and CAD/CAM retainers, using the impression replica technique. Only statistically significant sites are shown. Detailed results for all 12 measurement sites are provided in the Supplementary Material (Table S1).

Measurement region	Conventional retainers Median (IQR), mm	CAD/CAM retainers Median (IQR), mm	p-value
16A	0.068 (0.057–0.107)	0.111 (0.095–0.144)	0.003*
16B	0.158 (0.131–0.205)	0.142 (0.104–0.210)	0.411
13A	0.109 (0.080–0.136)	0.116 (0.087–0.140)	0.601
13B	0.289 (0.201–0.337)	0.180 (0.119–0.225)	0.008*
11A	0.178 (0.138–0.261)	0.116 (0.094–0.150)	0.009*
11B	0.271 (0.189–0.399)	0.187 (0.151–0.296)	0.030*
21A	0.186 (0.145–0.273)	0.108 (0.076–0.146)	< 0.001*
21B	0.255 (0.151–0.384)	0.164 (0.121–0.191)	0.009*
23A	0.141 (0.126–0.198)	0.085 (0.057–0.135)	0.007*
23B	0.184 (0.105–0.309)	0.129 (0.106–0.214)	0.073
26A	0.121 (0.101–0.179)	0.154 (0.099–0.232)	0.247
26B	0.148 (0.110–0.240)	0.173 (0.116–0.236)	0.940

*Significant difference ($p < 0.05$, Wilcoxon's signed-rank test).

Table S2. Comparison of the median thickness (gap) values at each measurement site between conventional and CAD/CAM retainers, using the 3D triple-scan protocol. Only statistically significant sites are shown. Detailed results for all 12 measurement sites are provided in the Supplementary Material (Table S2).

Measurement region	Conventional retainers Median (IQR), mm	CAD/CAM retainers Median (IQR), mm	p-value
16A	0.056 (0.035–0.075)	0.060 (0.027–0.158)	0.455
16B	0.182 (0.148–0.251)	0.119 (0.080–0.247)	0.113
13A	0.148 (0.138–0.222)	0.190 (0.138–0.281)	0.247
13B	0.386 (0.283–0.508)	0.337 (0.268–0.455)	0.765
11A	0.263 (0.205–0.316)	0.171 (0.104–0.303)	0.067
11B	0.372 (0.283–0.436)	0.376 (0.284–0.521)	0.538
21A	0.279 (0.232–0.401)	0.157 (0.066–0.273)	0.002*
21B	0.267 (0.216–0.424)	0.225 (0.197–0.386)	0.232
23A	0.176 (0.120–0.241)	0.071 (0.051–0.215)	0.028*
23B	0.207 (0.150–0.344)	0.213 (0.117–0.304)	0.478
26A	0.062 (0.026–0.104)	0.076 (0.019–0.114)	0.837
26B	0.150 (0.102–0.182)	0.163 (0.098–0.248)	0.641

*Significant difference ($p < 0.05$, Wilcoxon's signed-rank test).
